# Single-cell transcriptome atlas revealed liver immune features in biliary atresia

**DOI:** 10.3389/fped.2026.1995199

**Published:** 2026-10-16

**Authors:** Xuelai Liu, Zhuo Liu, Huan Wang, Yuqiang Chen, Mao Ye, Xu Li, Yuanyuan Geng, Xiaoyun Cui, Feng He

**Affiliations:** 1 Department of General Surgery, Capital Center for Children’s Health, Capital Medical University, Capital Institute of Pediatrics, Beijing, China; 2 Department of Biochemistry and Immunology, Capital Center for Children’s Health, Capital Medical University, Capital Institute of Pediatrics, Beijing, China; 3 Department of Pathology, Heilongjiang Hospital of Beijing Children’s Hospital, Capital Medical University, Harbin, China; 4 Department of Science and Technology, Capital Center for Children’s Health, Capital Medical University, Capital Institute of Pediatrics, Beijing, China

**Keywords:** biliary atresia, fibrosis, inflammation, ligand-receptor interaction, single cell sequence

## Abstract

**Background:**

While immune dysfunction is recognized as pivotal in biliary atresia (BA) pathogenesis, comprehensive characterization of disease-specific immune cell subtypes and stromal components remains incomplete. This study aims to define the altered hepatic immune-stromal landscape and aberrant intercellular communication networks associated with BA progression using single-cell transcriptomics.

**Methods:**

We performed single-cell RNA sequencing (scRNA-seq) on BA liver tissues to profile immune and stromal cell populations. Cellular subclustering, differential gene expression, pathway enrichment, and cell-cell communication analyses were employed to identify potential pathogenic drivers and predicted possible signaling networks. Multiplex immunofluorescence staining was used to verify receptor-ligand interaction in BA liver.

**Results:**

BA livers exhibited profound expansion of pro-inflammatory neutrophil subsets - Neutrophils with interferon-stimulated genes (Neu_ISG), Aged neutrophils (Neu_Aged) - with transcriptional signatures suggestive of enhanced reactive oxygen species (ROS) production, extracellular matrix (ECM) modulation, and cytokine activity. Concurrent cytotoxic/immunoregulatory lymphocyte populations - Effector memory CD4⁺ T Cell (CD4⁺ TEM), Tregs, cytotoxic NK cells- showed upregulated inflammatory and oxidative stress pathways. Periportal and lymphatic liver endothelial cells (LECs) demonstrated significant perturbations. Central venous LECs displayed signatures of oxidative stress and dysregulated proliferation/migration. Cell-cell communication analysis highlighted a possibly dominant “CXCL12-CXCR4” axis mediating neutrophil recruitment across endothelia, NK&T/B, and myeloid cells. Enhanced “MIF-CD74/CXCR4” signaling between endothelia and immune cells, “VEGFB-VEGFR1” interactions between hepatocyte-cholangiocyte compartments and central venous/periportal endothelia were observed, suggesting that these interactions are associated with BA progression.

**Conclusion:**

Our scRNA-seq atlas uncovers coordinated innate/adaptive immune responses and endothelial-stromal dysfunction that shape a hepatic niche in BA associated with ROS, unchecked inflammation, and fibrotic remodeling. Key cellular and molecular contributors include pathogenic neutrophils, cytotoxic lymphocytes, and injured endothelia, which interact via “CXCL12-CXCR4” and “VEGFB-VEGFR1” signaling axes. These findings from the present exploratory study point to potential novel therapeutic targets (e.g., neutrophil subsets, chemokine pathways) for mitigating BA pathogenesis.

## 1 Introduction

Biliary atresia (BA) is a severe inflammatory cholangiopathy in infants, which is characterized by intrahepatic and extrahepatic bile duct obstruction, liver fibrosis and eventually liver failure (1). Although Kasai operation may provide a relatively longer survival time for the autologous liver, the liver transplantation remains the only curative surgery for treating BA in most patients (2). Some reports have revealed that the pathogenesis of BA is related to dysplasia of ductal plate during fetal development (3), however, dysfunction of immune factors is considered to be main cause of the onset and development of BA (4). Recently, Wang et al. analyzed the immune characteristics of BA liver and reported comprehensive dysfunction of immune profiling by single-cell sequencing (5, 6). Although these findings verify the immune contribution to BA, the subtypes of immune cells that are integrally involved in BA have not been comprehensively analyzed. Importantly, stromal cells such as the endothelia cells in pathogenesis process of BA also needs to be taken seriously.

This study leverages scRNA-seq to define the altered immune and stromal landscapes in BA livers. We reveal a profound enrichment of pro-inflammatory neutrophil subsets within the BA hepatic microenvironment, including interferon-stimulated (Neu_ISG) and aged (Neu_Aged) subpopulations. These subsets exhibit signatures of enhanced reactive oxygen species (ROS) production, extracellular matrix (ECM) modulation (e.g., *COL17A1*, *VTN*), and cytokine activity (e.g., SLC25A34-AS1, *AGTR1*), suggesting a potential association between neutrophil activation and liver tissue inflammation and fibrosis in BA. Concurrently, we identify an expansion of cytotoxic and immunoregulatory lymphocytes—specifically CD4 + effector memory T cells (CD4 TEM), regulatory T cells (Tregs), and cytotoxic NK subsets—which demonstrate upregulated inflammatory pathways and oxidative stress responses. These findings suggest a coordinated immune response involving both innate and adaptive immunity.

Furthermore, we uncover significant perturbations in liver endothelial cells (LECs), particularly periportal liver sinusoidal endothelial cells (LSEc) and lymphatic ECs subtypes. Central venous LECs exhibit signatures of oxidative stress (*APOE*, *CCL2*) and dysregulated proliferation/migration (*NR2F2*, *EGFL7*), suggesting endothelial injury contributes to BA progression.

We further employ single-cell transcriptomics to characterize the aberrant intercellular communication networks within the BA hepatic niche. We reveal a prominent CXCL12-CXCR4 signaling axis that modulates neutrophil recruitment across multiple cell types (endothelial - NK&T/B cells/myeloid cells), highlighting this chemokine pathway as a key regulatory component in BA. Further, we also identify enhanced MIF-CD74/CXCR4 interactions between endothelia and immune populations-a pathway linked to macrophage survival and cytokine amplification. Notably, hepatocyte-cholangiocyte (HC) compartments showed pathological interactions with endothelia via VEGFB-VEGFR1 signaling, particularly with central venous/periportal subtypes, suggesting this pathway may mediate compensatory angiogenesis in response to vascular injury.

Collectively, our data provide a high-resolution atlas of the BA liver, identifying key cellular mediators—dysfunctional neutrophils, cytotoxic/activated lymphocytes, and injured endothelia—that orchestrate a microenvironment associated with ROS, unchecked inflammation, and fibrotic remodeling. These insights point to novel potential therapeutic targets for mitigating BA pathogenesis.

## 2 Materials and methods

### 2.1 Patients and tissue specimen collection

The specimen was collected at the Department of General Surgery, Capital Center for Children's Health, Capital Medical University between September 2023 and March 2024, including two BA liver tissues in patients confirmed by preoperative clinical presentation and biochemical examination, as well as the intraoperative observation and angiography, and one normal liver tissue adjacent to cancer in a hepatoblastoma patient confirmed by preoperative clinical presentation and CT examination, as well as the intraoperative operation. This study was approved by the Medical Ethics Committee of Capital Institute of Pediatrics (approval number: SHERLL2023023). Liver tissue was performed for the scRNA-seq and immunofluorescence staining. Tissue harvest method was same as our previous study. During the surgery, liver tissue from anterior margin of the left lobe of the liver for BA patients, and normal liver tissue adjacent to tumor mass for hepatoblastoma patient was harvested respectively. The tissue was divided into two parts, one part was put tissue preservation solution for scRNA-seq, and one part was put into 4% PFA solution for fixation and following histological study. Patients with BA and hepatoblastoma did not receive any other treatment prior to the surgery. While for the normal liver tissue adjacent to tumor mass for hepatoblastoma patient, we have confirmed its histological normalization, no inflammatory cells and tumor vascularization in it using histological examination before our study. We selected single-cell sequencing data for BA from public databases to perform cell subpopulation annotation and ligand-receptor interaction analysis between subpopulations, thereby comparing and validating our conclusions. The data were obtained from the Gene Expression Omnibus (GEO) database under accession number GSE176189. This dataset included 3 control samples (GSM5359607–GSM5359609) from liver tissues of pediatric patients with choledochal cyst and 9 BA samples (GSM5359592–GSM5359596, GSM5359610, GSM5359611, GSM5359622, GSM5359623).

### 2.2 Single-cell RNA sequencing data processing and analysis

Liver biopsies obtained during Kasai operation were collected in FACS buffer (PBS with 0.5% BSA, 1 mM EDTA, pH 8.0). After histological sampling, remaining tissue was washed three times with FACS buffer, transferred to a 70 μm strainer pre-wetted with 2 mL buffer, cut into <1 mm^3^ pieces, and mashed with a syringe plunger. The strainer was rinsed with an additional 2 mL buffer, and the suspension centrifuged at 1800rpm for 5 min. The pellet was resuspended in 1–2 mL red blood cell lysis buffer, incubated for 5 min at room temperature, then neutralized with 10 mL FACS buffer and centrifuged. Finally, cells were resuspended in FACS buffer. 5’ libraries for single-cell RNA sequencing were conducted with Chromium Single Cell 5’ Kit v2 (10× Genomics) according to the instructions from the manufacturer. Single-cell RNA sequencing was performed using Illumina Novaseq 6000 sequencer. Raw sequencing data were processed using the Cell Ranger pipeline (v5.0.0, 10× Genomics) to perform cellular barcode demultiplexing, align reads to the GRCh38 human reference genome via the STAR aligner, and generate a unified gene-cell unique molecular identifier (UMI) count matrix through read down-sampling across samples (7).

Subsequent analyses were conducted using the Seurat package (v3.1.1) in R. Quality-control filtering was performed to exclude low-quality cells based on gene expression complexity, mitochondrial transcript proportion, and abnormal transcript counts. Within each sample, cells with fewer than 200 or more than 10,000 detected genes, or with mitochondrial RNA content exceeding 10%, were filtered out based on quality control criteria. Genes detected in only a limited number of cells were excluded from downstream analyses. After quality-control filtering, a total of 24,882 cells were retained for subsequent analyses, including 17,608 cells from biliary atresia (BA) samples and 7,274 cells from control liver tissue (7).

Data normalization was performed using the NormalizeData function with the “LogNormalize” method, in which gene expression values for each cell were normalized by total expression, multiplied by a scaling factor of 10,000, and log-transformed. Highly variable genes (HVGs) were identified using Seurat function “FindVariableFeatures”, and the top 3,000 HVGs were selected for downstream analyses (8).

Dimensionality reduction was performed using principal component analysis (PCA) based on scaled HVG expression matrices. Harmony (version 1.2.4) was used to correct for batch effects, with parameters adjusted to nclust = 50, max_iter = 20, and early_stop = TRUE to ensure convergence. To identify distinct cell types, the ‘FindNeighbors’ function of Seurat was used to construct the SNN Graph, based on unsupervised clustering performed with “FindClusters” function with a resolution set to 0.5 (7). Two-dimensional visualization of cellular distributions was generated using t-distributed stochastic neighbor embedding (t-SNE) (7).

Cluster-specific marker genes were identified using the FindAllMarkers function in Seurat based on differential expression analysis between each cluster and all remaining cells. Cell-type annotation was performed according to canonical marker genes reported in previous studies and established lineage-specific signatures (6, 9, 10).

Differentially expressed genes (DEGs) between BA and control groups or among cellular subpopulations were analyzed using the statistical framework implemented in Seurat. Genes with an absolute log₂ fold change (|log₂FC|) ≥ 0.25 and an adjusted *P* value (Padj) ≤ 0.05 were considered statistically significant and retained for downstream analyses (7). Given the limited number of biological samples, these DEG analyses were performed at the single-cell level and should be interpreted as exploratory rather than as patient-level inferential statistics.

### 2.3 Pathway enrichment analysis

Pathway enrichment analyses were performed using the clusterProfiler R package together with the org.Hs.eg.db annotation database. Gene Ontology (GO) and KEGG enrichment analyses were conducted, and adjusted *p* value < 0.05 were calculated using the Benjamini–Hochberg false discovery rate (FDR) correction method (11).

### 2.4 Cell-cell communication inference

Cell-cell communication analysis was performed using CellChat (v1.1.3) to infer ligand-receptor interaction networks among different cellular subpopulations. Communication probability and interaction strength were visualized using the netVisual_heatmap and netVisual_bubble functions (12).

### 2.5 Gene set scoring at single-cell resolution

Gene set scoring analyses were conducted at single-cell resolution using previously published inflammatory-response, cytokine-response, and interferon-related gene signatures. Relative activity scores among different cell populations were compared to evaluate immune activation states and inflammatory characteristics. The “AddModuleScore” function in Seurat was used to implement the method with default settings.

All downstream statistical analyses and data visualization were performed using R software (13). Given the small number of biological samples (2 BA and 1 control), comparisons of cell-type proportions were descriptive, and no patient-level statistical testing was performed.

### 2.6 Multiplex immunofluorescence staining

To validate the ligand-receptor interactions between cell subpopulations, we performed multiplex immunofluorescence assays. In addition to the liver tissues from the two included pediatric BA patients and one control sample, we additionally selected liver tissues from another two BA patients and another paracancerous normal liver tissues from three pediatric patients with hepatoblastoma for multiplex immunofluorescence staining validation.

The liver tissue from the pediatric patient obtained via surgical resection was processed using formalin-fixed paraffin-embedding (FFPE) and sectioned into 4 μm thick slices. Four-plex immunofluorescence staining was sequentially performed. Following deparaffinization and rehydration through a graded ethanol series, heat-induced antigen retrieval was conducted using EDTA buffer (pH 9.0). Endogenous peroxidase activity was quenched with a 3% hydrogen peroxide solution, and non-specific binding sites were blocked with 10% normal goat serum at room temperature.

The staining employed a tyramide signal amplification (TSA)-based sequential labeling approach. For the first round, sections were incubated with a rabbit primary antibody at 4 °C overnight, followed by an HRP-conjugated anti-rabbit secondary antibody at room temperature. Signal amplification was then achieved using a Cy3-conjugated tyramide reagent. Subsequently, the residual HRP activity was inactivated by treating the sections with a buffer containing catalase to terminate the first-round reaction.

The subsequent two rounds of staining were iteratively performed following the aforementioned procedure, respectively, along with their corresponding HRP-conjugated secondary antibodies and Cy5 or other fluorescent dye-conjugated tyramides. Upon completion of all staining rounds, cell nuclei were counterstained with DAPI. The slides were scanned using a Olympus VS200 slide scanner. Multichannel fluorescence images were acquired with the OlyVia software. Information of the primary antibody were listed in Table 1.

**Table 1:** Primary monoclonal/polyclonal antibodies used in this study.

Abbreviation	Dilution	Species	Manufacturer	Catalog numbers
CD3	1:1,000	Rabbit	Proteintech	17617-1-AP
CK19	1:6,000	Rabbit	Proteintech	10712-1-AP
CD68	1:100	mouse	Proteintech	66231-2-Ig
CD31	1:1,000	Rabbit	Proteintech	11265-1-AP
CXCL12	1:200	Rabbit	Proteintech	17402-1-AP
CXCR4	1:600	mouse	Proteintech	60042-1-Ig
VEGFB	1:600	mouse	ZSGB-BIO	TA507205
VEGFR-1	1:1,000	Rabbit	Proteintech	13687-1-AP

For multiplex immunofluorescence quantification of CXCL12–CXCR4 interactions between cells, such as T cells and endothelial cells, 4 biological samples per group (BA: *n* = 4; control: *n* = 4) were analyzed, with 5 random fields of view (FOVs) per sample, yielding 20 FOVs per group. The interaction index was defined as the number of adjacent CXCL12⁺ endothelial cell–CXCR4⁺ T cell pairs per field of view, with adjacency defined as direct membrane contact. Statistical comparisons were performed using the Mann–Whitney U test (two-tailed), with each FOV treated as an independent observation. A *P* value < 0.05 was considered statistically significant.

## 3 Results

To comprehensively uncover the cellular and molecular pathology underlying BA, we employed droplet-based scRNA-seq to analyze the transcriptomic profiles of liver from 2 BA children and 1 normal tissue adjacent to tumor of children with hepatoblastoma. A total of 24,882 cells successfully passed quality control. These included 17,608 cells from BA and 7,274 from CON. Ten major cell types were clustered based on the expression of canonical marker genes, namely B cell, intrahepatic biliary epithelial cells (BEC), endothelia (Endo), erythroblast, fibroblast, hepatocyte and cholangiocyte (HC), mast cell, mononuclear phagocytes (MP), neutrophils and NK&T (NK and T cell) (Figures 1A,B). To unveil distinctive immune profiles of BA and control group, cell composition was then analyzed descriptively (Figure 1C). Figure 1D showed characterization of cell subsets in BA by marker gene expression and immune-related scores. The results showed that the proportions of B cells and HC appeared higher in BA, consistent with previous report (5, 14). The proportions of endothelia, NK&T cells, and neutrophils were comparable between the BA and control groups, as previous studies have implicated these cell types in BA pathogenesis, we then focused on these cell types (14, 15).

**Figure 1:**
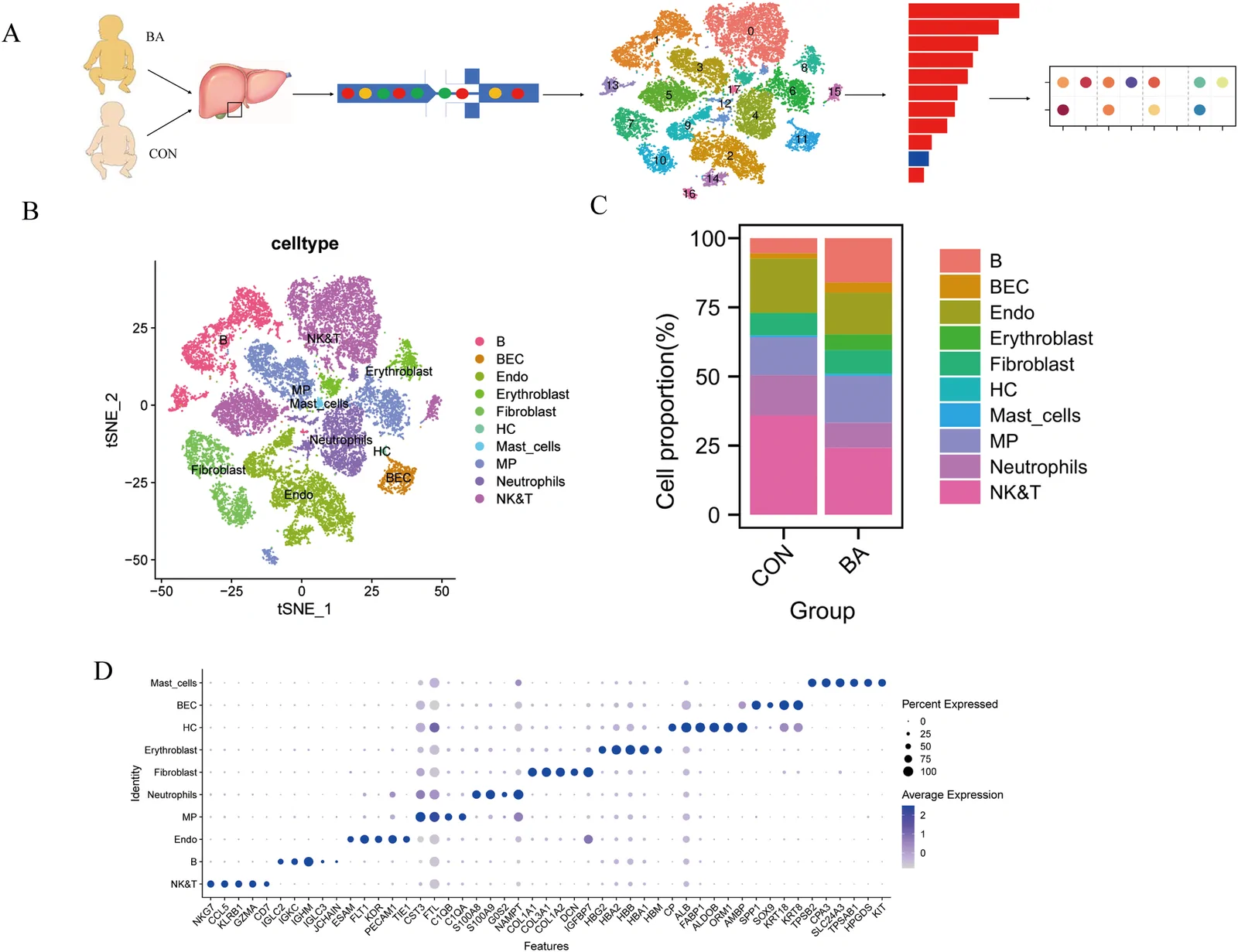
Human liver cell atlas for biliary atresia by single-cell transcriptome. **(A)** Schematic diagram showing the over pipeline for 10× liver scRNA-seq (*N* = 2 for BA and *N* = 1 for control) followed by bioinformatic analysis of cell cluster analysis, GO analysis and cell-interaction analysis. **(B)** t-SNE plot showing cells colored by major cell types. **(C)** Stacked plot showing proportion of the major cell types of BA and CON group. **(D)** Marker genes and immune related scores of cell subsets in BA.

### 3.1 Enrichment of immune response and higher ROS level in neutrophils in BA

Neutrophils were analyzed and sub-clustered into 7 substantial heterogeneous subtypes, including one mature subset (Neu_Mature_02), three homeostatic subsets (Neu_Homeostatic_01, Neu_Homeostatic_02 and Neu_Homeostatic_03), two interferon-stimulated genes (ISG)-related subsets (Neu_ISG_01 and Neu_ISG_02), and one aged subset (Neu_Aged_01) (Figure 2A), marker genes were shown (Supplementary Figure S1A). All of the neutrophil subsets were present in both healthy controls and BA children, while their proportions were different (Figure 2B).

**Figure 2:**
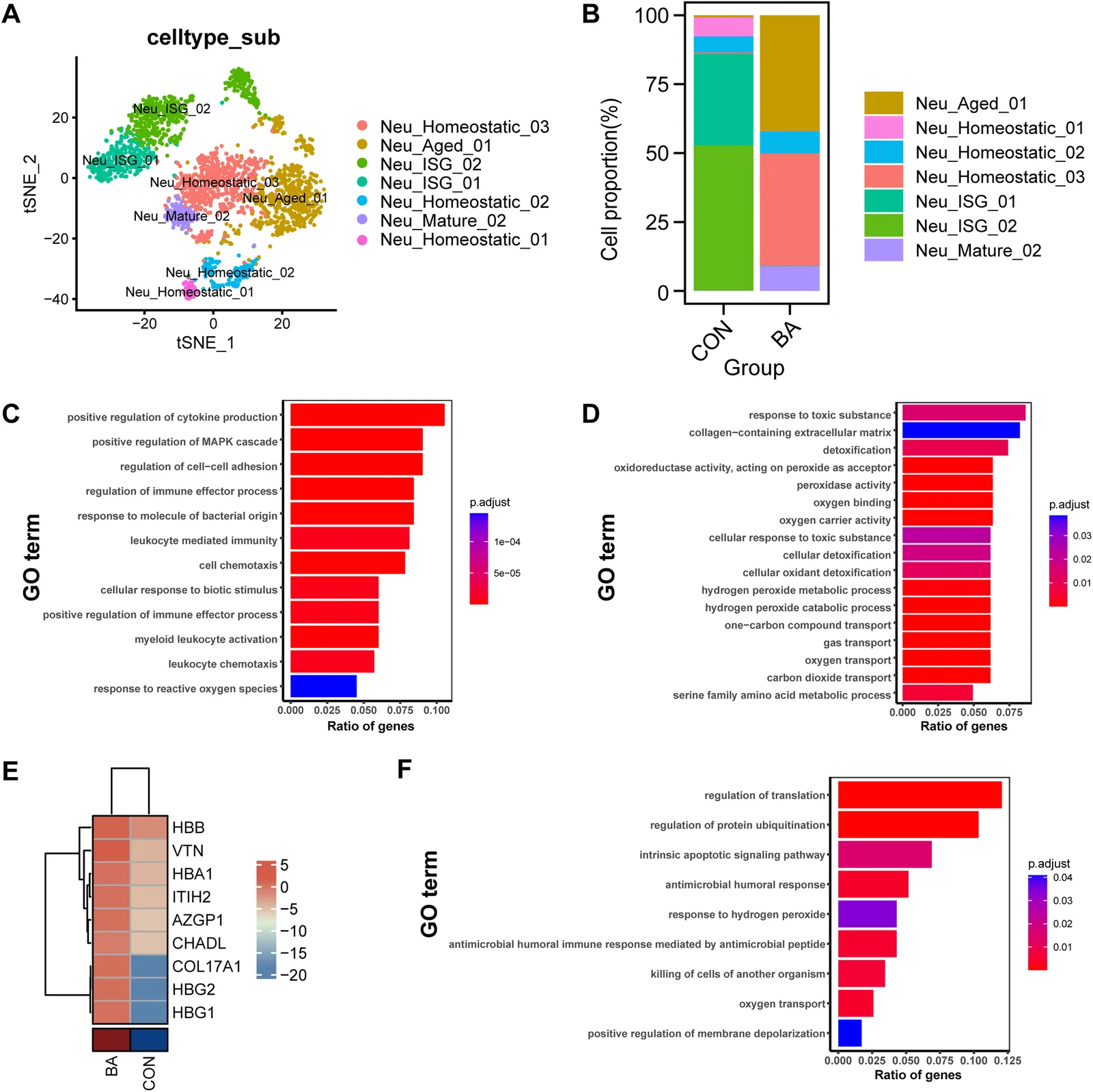
Analysis of neutrophils reveals upregulated immune response and oxidative stress response in BA. **(A)** t-SNE plot showing cells colored by subtypes of neutrophils. **(B)** Stacked plot showing proportion of the subtypes of neutrophils in BA and CON group. **(C)** Barplots showing enriched biological processes for upregulated genes in neutrophils in BA. Color indicates *p*-value. **(D)** Barplots showing enriched biological processes for upregulated genes in neutrophil ISG subtype in BA. Color indicates *p*-value. **(E)** Expression of collagen and ROS related genes in BA and CON groups. Color indicates relative expression of genes. Color scale represents normalized mean gene expression level. **(F)** Barplots showing enriched biological processes for upregulated genes in neutrophil homeostatic subtype in BA. Color indicates *p*-value.

The neutrophil extracellular trap (NET)-forming neutrophils resulted in cholangiocyte injury in BA, indicating that neutrophil play a functional role in BA pathogenesis (15, 16). In the fibrosis area of nonalcoholic steatohepatitis, neutrophils expressing MMP9, which participates in the degrading extracellular matrix including collagen, were accumulated and participates in the fibrosis process (17), suggesting that neutrophils may play a role in fibrosis modulation in BA. To explore functional difference between BA and control group, differently expressed genes (DEGs) analysis of neutrophils were analyzed. Gene ontology (GO) enrichment analysis was conducted and were found enriched for terms to immune response and neutrophil function, containing “positive regulation of cytokine production”, “cell chemotaxis”, “regulation of immune effector process” and “cellular response to biotic stimulus”, indicating that neutrophil in BA exert its cytokine regulation and immune regulation function (Figure 2C).

Oxidative stress is an important cause of fetal injury (18), exogenous hydrogen peroxide exacerbate neutrophil recruitment and expression of neutrophil-active chemokine in liver and induce liver cell necrosis (19). We found that differently expressed gene in neutrophil also present “response to reactive oxygen species” function, which suggests that reactive oxygen species (ROS) may contribute to the pathogenesis of BA. Further, SLC25A34-AS1, which belongs to acute phase proteins, was elevated in Neu_ISG_01 subset, indicating that Neu_ISG_01 subset contributed to the modulation of inflammation in BA group (Supplementary Figure S1B). Interestingly, AGTR1 gene, which was proved to be associated with immune activation (20), was elevated in Neu_ISG_01 subset in this study, which may contribute to activation of Neu_ISG_01 subset (Supplementary Figure S1C). These results indicated that neutrophils in BA display pro-inflammatory properties and exhibit transcriptional features associated with ROS production, in line with prior reports (21, 22).

We next focused on the functional role of Neu_ISG subtype. GO enrichment analysis of Neu_ISG subtype revealed that the upregulated genes were enriched for terms of “oxygen binding”, “peroxidase activity” and “oxygen transport” (Figure. 2D), which were compatible with previously studies that neutrophils showed increased levels of reactive oxygen species in biliary atresia (15). Interestingly, we observed an enrichment of “collagen-containing extracellular matrix” term in Neu_ISG subset according to the elevated genes, such as COL17A1 that related with fibrosis, and ITIH2, CHADL and VTN that was associated with extracellular matrix modulation process (Figure 2E).

In the BA group, Neu_Aged subtype was elevated in the liver (Figure 2B), these subsets were characterized by elevated expression of age-related genes like CXCR4, PI3 and SIGLEC10 (9, 23). Neu_Aged cell subtype derived from the circulation in bone marrow, liver, and spleen and exhibited high phagocytic activity in acute inflammatory response (24), Neu_Aged cell could also induce CD4 ^+^ T cell to aggravate inflammation and cause neutrophil extracellular traps (NET) (25). Here our results showed that proportion of Neu_Aged cell were much higher in BA than control group, indicating that aged neutrophils in BA may be associated with the inflammatory processes in BA. GO enrichment analysis of Neu_homeostatic cell type in BA group also showed function of “response to hydrogen peroxide”, “oxygen transport” etc. (Figure 2F), which were similar to Neu_ISG subtype.

We calculated an inflammatory score and IFN score for each subset of neutrophil cell. Results revealed a noticeable decrease in the expression of IFN related genes and higher inflammatory score in the homeostatic 03, aged 01 and mature02 subclusters, indicating these subsets were associated with inflammation process, (Supplementary Figures S1D,E).

### 3.2 Upregulated inflammatory response and cytotoxicity in NK and T cell in BA

Further clustering of NK&T cells yielded 9 subclusters, including CD4 naïve, CD4 Treg, CD4^+^ effector memory, CD8 naïve, CD8 effector memory, CD8 experience, NK resting, NK cytotoxic and proliferating NK&T(Pro-NK&T). Higher proportion of CD4^+^ effector memory T cell, CD4^+^ Treg were found in BA group (Figures 3A,B).

**Figure 3:**
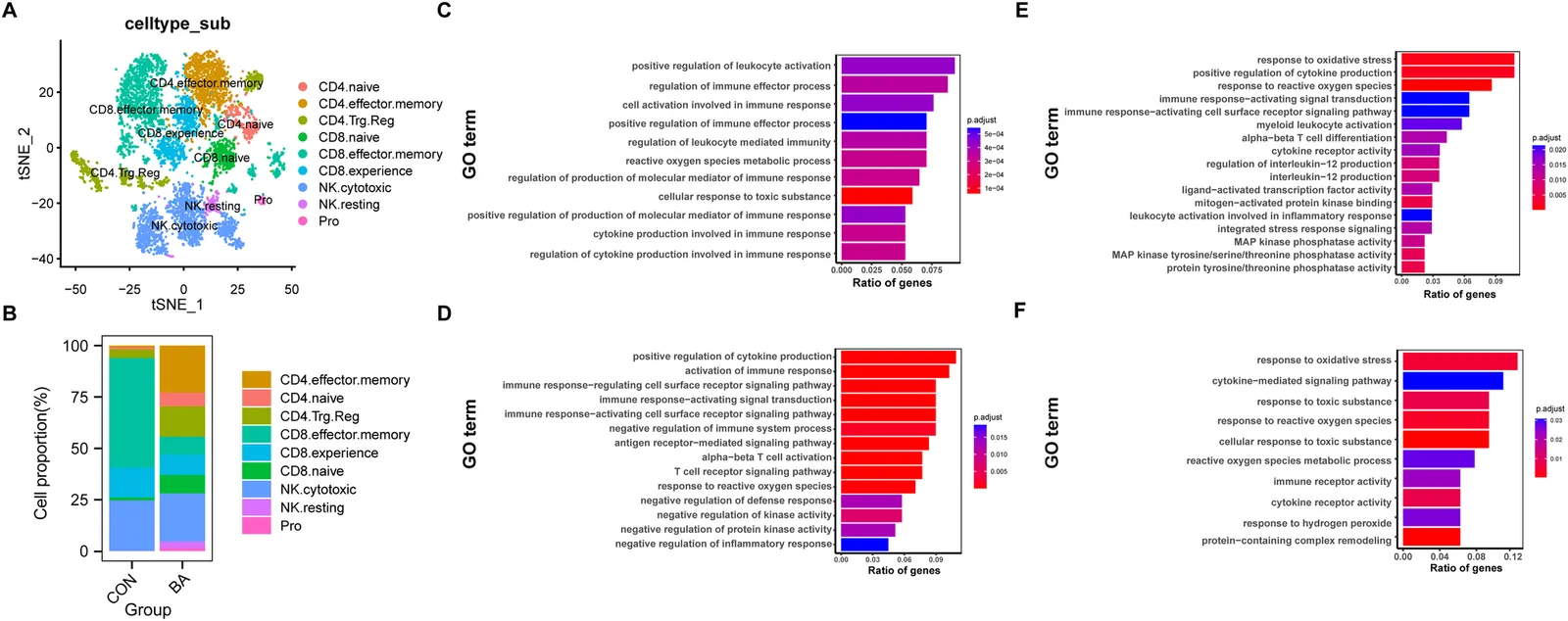
Analysis of NK&T cell reveals upregulated inflammatory response and cytotoxicity in BA. **(A)** t-SNE plot showing cells colored by subtypes of NK&T. **(B)** Stacked plot showing proportion of the subtypes of NK&T in BA and CON group. **(C)** Barplots showing enriched biological processes for upregulated genes in CD4_effector memory in BA. Color indicates *p*-value. **(D)** Barplots showing enriched biological processes for upregulated genes in CD4 Treg in BA. Color indicates *p*-value. **(E)** Barplots showing enriched biological processes for upregulated genes in CD8 experience subtype in BA. Color indicates *p*-value. **(F)** Barplots showing enriched biological processes for upregulated genes in NK cytotoxic subtype in BA. Color indicates *p*-value.

The inflammatory response plays a significant role in the development of BA (26), while the origins of cytokine production in BA patients remain unclear. To investigate the potential sources of cytokine genes, we systematically analyzed the expression of established inflammatory response genes and a set of cytokine genes (9, 13, 27). We calculated an inflammatory score and a cytokine score for each subset of NK&T cell based on the expression levels of these genes. Results showed that IFN related genes were downregulated in BA group, and a noticeable increase in the expression of inflammatory and cytokine genes in NK_resting and Pro-NK&T subtype in BA compared to CON (Supplementary Figures S2B,C,D), suggesting the presence of inflammatory response in BA group.

CD4^+^ effector memory T (CD4 T_EM_) subset were characterized by elevated expression of IL7R, CD40LG, ANXA1, FOS and JUN. CD4 T_EM_ derives from CD4T cells and play a critical role in the adaptive immune system and were characterized by rapidly producing TNF-α or IFN-*γ* and mediate inflammation in early life (28). In the BA group, proportion of CD4 T_EM_ were much higher than control group, indicating CD4 T_EM_ exerting its function in the process of BA. We also found that process of “positive regulation of leukocyte activation”, “regulation of immune effector process”, “positive regulation of immune effector process” and “regulation of leukocyte mediated immunity” were enriched in CD4 T_EM_ (Figure 3C), indicating that CD4 T_EM_ may contribute to severe inflammatory response in BA pathogenesis.

CD4 Treg subset were characterized by elevated expression of FoxP3, SAT1, IL2RA and CTLA4 (Supplementary Figure S2A). Treg functions as immune-negative modulator. Treg could secrete inhibitory cytokines, exert direct contact dependent inhibitory effects, and downregulate antigen-presenting cell (APC) function (29). Here, proportion of Treg in the liver was elevated in BA, which was constant with previous study (30). GO analysis revealed that “positive regulation of cytokine production”, “activation of immune response”, “alpha-beta T cell activation” and “T cell receptor signaling pathway” were enriched in Treg in BA (Figure 3D). Previous study proved that Treg from peripheral blood from patients with BA had activated phenotype (31), which was in accordance with our result.

Proportion of CD8^+^ effector memory T (CD8 T_EM_) subset were shown to be decreased in BA group. CD8T_EM_ subset originate from CD8T cells and exert an initial immune response, CD8 T_EM_ cells have memory characteristics that enable them to quickly recognize and respond upon re-exposure to the same pathogen (32). We compared inflammatory scores, cytokine scores and IFN scores for NK&T cell subsets (Supplementary Figures S2B,C,D), surprisingly, IFN scores were lower in NK&T cell subsets, specifically, IFN-stimulated genes (ISG) such as IFIT1, MX2, OAS1, STAT1, MX1, IFI6 and ISG15 were downregulated in CD8 T_EM_, providing a critical mechanistic insight into the inactivation of interferon response of CD8 T_EM_ cells (Supplementary Figure S2E). Previous study indicate that upregulation of inhibitory T cell receptors on T_EM_ cells inhibits its immune response and potentially limits T cell-mediated liver damage (33), the downregulated ISG related genes in CD8 T_EM_ indicate that immune suppression state of T_EM_ in BA exert its protection function.

ROS have emerged as important modulators of T cell proliferation (34), upon antigen presentation, ROS are needed to achieve TCR signaling while unlimited ROS may cause cell necrosis and elicit severe inflammation and damage (35). Here, terms such as “response to oxidative”, “response to reactive oxygen species”, and “integrated stress response signaling” were enriched in CD8_experience cells using GO analysis, suggesting oxidative stress response showed an increase and associated with severe inflammation in BA (Figure 3E).

NK cells is a kind of cytotoxic lymphocytes that are capable of triggering granzyme-mediated death of target cells, and may exert its function of killing fibroblasts (5, 36). Here, NK cells were stratified into two subsets, NK_cytotoxic and NK_resting. GO analysis showed that “response to toxic substance”, “cellular response to toxic substance” were enriched in NK cells, further, “cytokine mediated signaling pathway”, “response to oxidative stress” and “cytokine receptor” (Figure 3F), implying that NK cell in BA exhibit pro-inflammatory characteristics.

### 3.3 Enhanced chemotaxis of endothelia in BA

Liver endothelial cells (LEC) are an important type of cell in the liver. LEC act as a “filter” to regulate metabolite transportation from the intestine to liver cells, and could also control immune response to viral infections independent of leukocyte (37), this indicates that LEC has an important guiding role in the process of maintaining liver stability (38). LECs could also secret different chemokines to recruit immune cells from circuiting blood to tissues**.** Here, endothelial cells were analyzed and sub-clustered into 4 heterogeneous distinct subtypes, including periportal liver sinusoidal endothelial cells (LSEC), central venous LSEC, veins endothelial cells (EC) and lymphatic ECs (Figure 4A). A bubble graph showing marker gene expression in each endothelial cell subtypes (Supplementary Figure S3). Within these 4 subclusters, proportion of periportal LSEC and lymphatic ECs were much higher than control groups (Figure 4B), which were in accordance with the result of previous study (39, 40).

**Figure 4:**
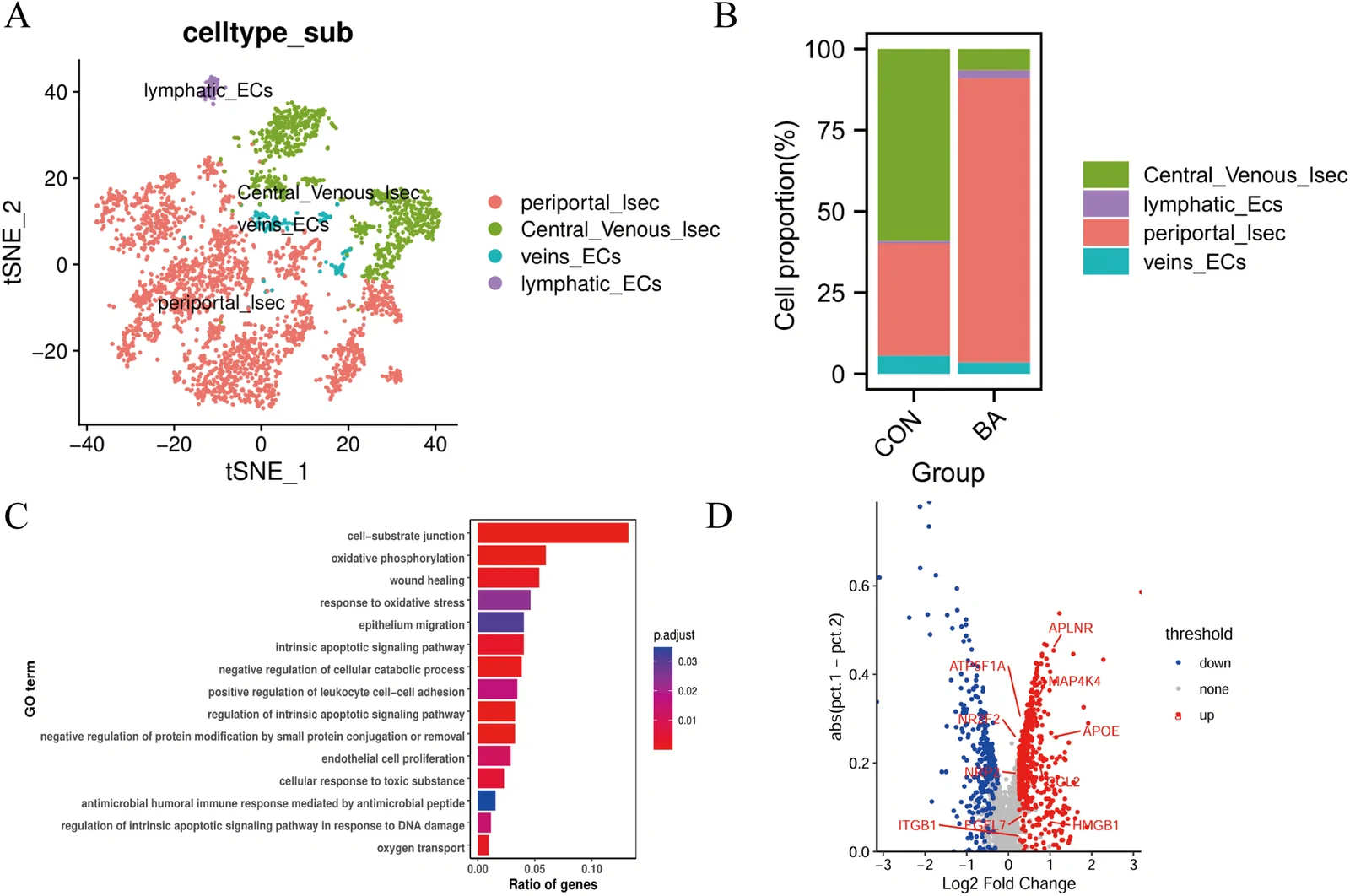
Endothelial cell subtype distribution and oxidative stress-related functional changes in BA. **(A)** t-SNE plot showing cells colored by subtypes of endothelial cells. **(B)** Stacked plot showing proportion of the subtypes of endothelial in BA and CON group. **(C)** Barplots showing enriched biological processes for upregulated genes in central venous in BA. Color indicates *p*-value. **(D)** Volcano plot showing specific genes expression in central venous LSEC in BA.

To delineate the function of periportal in the BA group, GO analysis of differently expressed genes were conducted. The upregulated genes in central venous LSEC were enriched in the process associated with apoptosis (Figure 4C), these findings indicated that central venous LSEC were injured in BA, compared with that of control group. Previous study proved that redox stress in liver tissue is a negative prognostic factor in patients with BA (41), here, we found central venous LSEC showed high evidence of oxidative stress, because term of “oxidative phosphorylation”, “oxygen transport” and “response to oxidative stress” were enriched in the differently expressed genes (Figure 4C). Further, “endothelial cell proliferation and migration”, and “negative of regulation of protein modification” were also enriched in the LSEC of BA, genes associated with “endothelial cell proliferation” such as APOE/MAP4K4/CCL2/ITGB1/HMGB1 and “epithelium migration” such as “NR2F2/NRP2/EGFL7/APLNR/ATP5F1A” were elevated in BA (Figure 4D), all of the processes may attribute to the inflammation and cholestasis of BA.

### 3.4 Endothelia interact with other cells in BA

Biliary atresia was characteristic by thickening of the extrahepatic portal vein wall, with the main pathological changes being infiltration of large numbers of inflammatory cells, to investigate the potential crosstalk of endothelial and immune cells specifically may be specific to BA, cell-cell interaction analysis was conducted. The result showed preferential interactions between neutrophil and other cell subsets in BA group (Figure 5A). We further explored the ligand-receptor (L-R) pairs mediating these interactions. The results showed that CXCL12-CXCR4 showed a strong potential interaction capacity between endothelial and other immune cells, such as NK&T, B, Mp and neutrophil (Figure 5B), indicates that this pair is widely distributed and may reflect the importance of chemotaxis function of endothelial by CXCL12 in BA and may contribute to the pathogenesis. The CXCL12/CXCR4 axis has an important role in a broad range of disease, in myocardial infarction, neutrophils drove excessive release platelets by CXCL12/ CXCR4 axis and increase the risk of recurrent ischemia (42). In hepatocellular carcinoma, CXCL12/CXCR4 mediate B cell recruitment and plasma cell differentiation and has a role in inhibiting tumor (43). These results illustrate that the ligand-receptor interaction may play a key role in the pathogenesis process in BA. We specifically confirmed CXCL12-CXCR4 pairs between endothelia vs. mononuclear phagocyte, endothelia vs. T cell and cholangiocyte vs. T cell in BA patients (Figures 5C,E,G). We also selected an increased number of BA samples and normal samples, and statistically compared the CXCL12-CXCR4 pairs positive areas in randomly chosen low-magnification fields, results showed the number of positive areas was higher in BA group (Figures 5D,F,H). Another L-R pair of MIF-CD74/CXCR4 pair was also found exist between endothelial and other immune cells, such as NK&T, B, Mp and neutrophils (Figure 5B). Macrophage migration-inhibitory factor (MIF) is an atypical chemokine, the MIF/CXCR4 pairs were proved to contribute to atherosclerosis by mediating arterial leukocyte adhesion, enhancing cell survival and cytokines secretion (44). MIF-2, a paralog of the cytokine MIF, could promote hepatic lipid accumulation (45). MIF-2 inducible CD74/CXCR4 complexes activates sterol regulatory element-binding protein (SREBP)-driven lipogenic genes by leukocyte migration and endothelial arrest and promotes atherosclerosis (45). Our findings demonstrate that the MIF-CD74/CXCR4 axis may contributes to the progression of BA by regulating immune cell chemotaxis. To validate the important role of these interactions in BA samples, we collected single-cell sequencing data of BA from public databases and performed ioinformatics analysis and cell clustering (Supplementary Figures S4A,B,C), followed by analysis of MIF-CD74/CXCR4 intercellular interactions among cell subpopulations. We found that these interactions also widely exist between non-parenchymal cells and immune cells, highlighting their critical significance (Supplementary Figure S4D). It should be noted that the differences in the UMAP spatial distribution of cell subpopulations between our single-cell data and the public dataset may be attributed to differences in sequencing platforms, experimental batches, sample processing protocols, as well as potential variations in disease stages of the samples.

**Figure 5:**
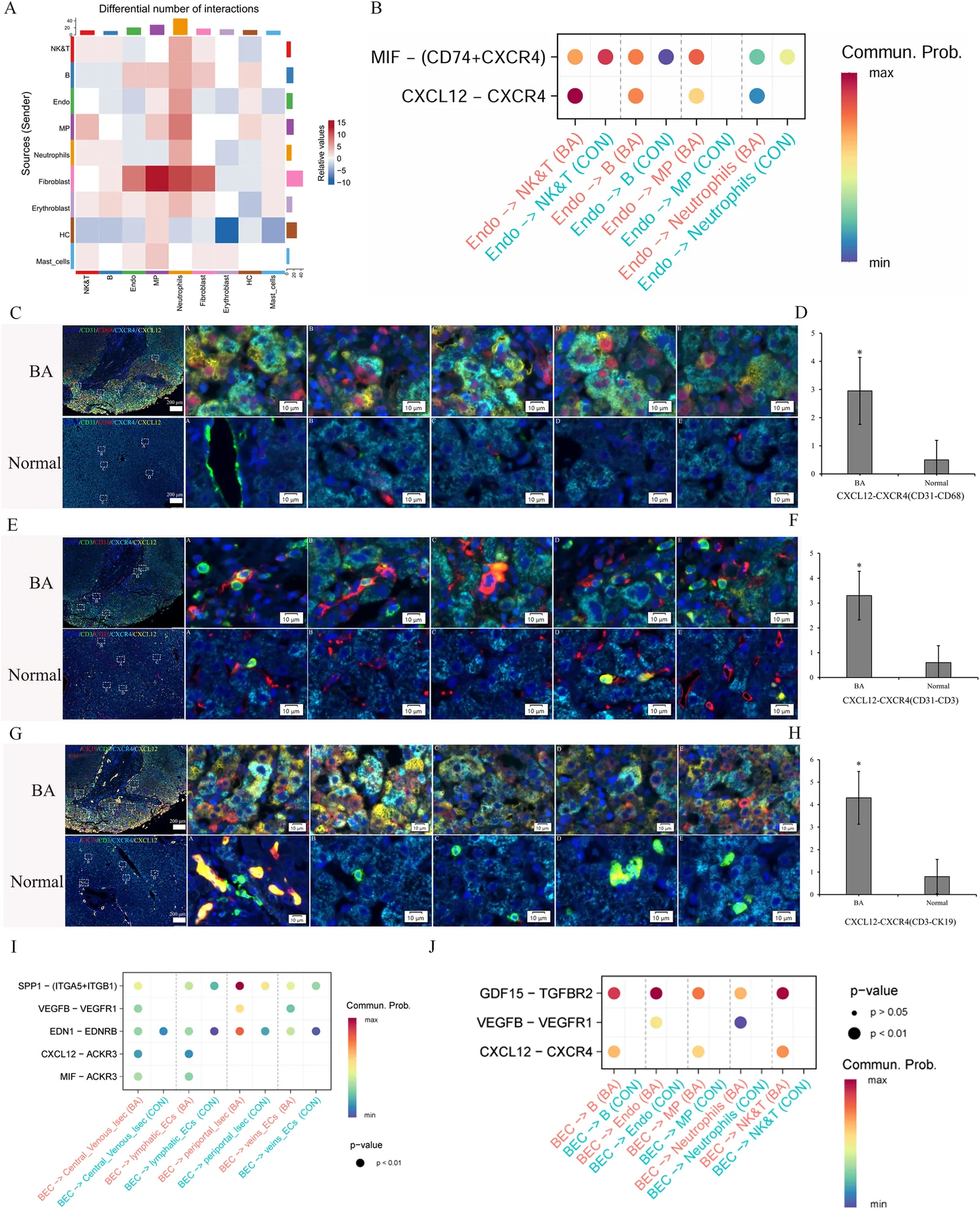
Cell-cell interaction analysis between HC, endothelial cells and immune cells. Multiplex immunofluorescence was used to detect ligand-receptor interactions between cell subpopulations. For the two pediatric BA patients and one control included in the single-cell sequencing analysis, we additionally incorporated liver tissues from two other pediatric BA patients and paracancerous normal tissues from three pediatric patients with hepatoblastoma for immunofluorescence detection. Subsequently, statistical analysis was performed on the positively stained regions across multiple tissue samples. **(A)** Intercellular communication network among various cellular subpopulations. **(B)** Spotplots of ligand-receptor interaction between endothelial (as ligand) and subtypes of immune cells (as receptor) between BA (red in x-axis) and CON (blue in x-axis). **(C)** Multiplex immunofluorescence showed CXCL12-CXCR4 expression in endothelial cells and MP in BA and control liver tissues. **(D)** Statistical result of number of CXCL12-CXCR4 pairs between endothelial and MP in randomly chosen low-magnification fields of liver section of BA group and control group (*N* = 4, 5 random FOVs per sample). The vertical axis represents the average number of CXCL12-CXCR4 pairs per low-magnification field. **(E)** Multiplex immunofluorescence showed CXCL12-CXCR4 interactions between vascular endothelial cells and T cell. **(F)** Statistical result of number of CXCL12-CXCR4 pairs between vascular endothelial cells and T cell in randomly chosen low-magnification fields of liver section of BA group and control group (*N* = 4, 5 random FOVs per sample). The vertical axis represents the average number of CXCL12-CXCR4 pairs per low-magnification field. **(G)** Multiplex immunofluorescence showed CXCL12-CXCR4 interactions between cholangiocyte and T cell. **(H)** Statistical result of number of CXCL12-CXCR4 pairs between cholangiocyte and T cell in randomly chosen low-magnification fields of liver section of BA group and control group (*N* = 4, 5 random FOVs per sample). The vertical axis represents the average number of CXCL12-CXCR4 pairs per low-magnification field. **(I)** Spotplots of ligand-receptor interaction between BEC (as ligand) and other types of endothelial cells (as receptor) between BA (red in x-axis) and CON (blue in x-axis). Color represents the communication probability of ligand-receptor pairs. **(J)** Spotplots of ligand-receptor interactions between BEC (as ligand) and immune and endothelial cell populations (as receptor) in BA and CON groups. Abbreviations: Endo, endothelial; NK&T, NK and T cell; B, B cell; MP, mononuclear phagocyte; HC, hepatocyte and cholangiocyte; BEC, biliary epithelial cells; BA, biliary atresia; CON, control group.

In our results, ten major cell types were clustered based on the sequence of the whole liver tissue, HC subset contains two cell subutypes, including hepatocytes and cholangiocytes. Hyperplasia of cholangiocytes is an important characteristic of biliary atresia. Interaction between HC and endothelial were analyzed. Interaction such as SPP1-(ITGA5+ITGB1), VEGFB-VEGFR1, EDN1-EDNRB, were found to be widely exist between HC and endothelial and interaction of CXCL12-ACKR3 and MIF-ACKR3 were found between HC and central venous/lymphatic EC. Specially, EDN1-EDNRB interaction were proved exist between non-parenchymal cells in the BA patients from public databases (Supplementary Figure S4E). It should be noted that interaction between VEGFB on BEC cells and VEGFR1 on endothelial may functions in BA pathogenesis (Figure 5I), vascular endothelial growth factor (VEGF) superfamily consists of several members, VEGF-A, VEGF-B, VEGF-C, VEGF-D and planta growth factor (PLGF), which can promote increased cell proliferation, angiogenesis, inflammation and participate in multiple angiogenesis associated disease. BA may cause severe damage to blood vessels in the liver, the VEGFB/VEGFR1 pair in BA may functions in the process of BA. We further analyzed the pair between HC and all of the subtype of endothelial. VEGFB/VEGFR1 pair showing increased interactions between HC and central Venous/periportal/veins, indicating in the progress of BA, HC may be modulated by endothelia. Taken together, these data demonstrate that VEGFB/VEGFR1 is an important signaling pathway augmented in BA cholangiocytes.

We next explore interaction of BEC and immune cell. Analysis indicated that GDF15-TGFBR2, VEGFB-VEGFR1 and CXCL12-CXCR4 pairs exist in BA group (Figure 5J), specially, GDF15-TGFBR2 were widely distributed between HC and endothelial/all of the other immune cell, including NK&T, B, Mp and neutrophils. GDF15-TGFBR2 reside between macrophage and CD8 exhausted T cell and play a key immunosuppressive role in cancer (46), this imply that this pair may exert immunosuppressive function in BA process.

Taken together, we characterized the immunocyte interactions in immune cell and hepatic parenchymal cells and other non-parenchymal cells at a single-cell scale and specially identified the presence and functional relevance of CXCL12-CXCR4 pair in BA.

## 4 Discussion

In this report, we applied scRNA-seq to create a landscape of the immune microenvironment and characteristic of endothelial cells regarding BA. Although single cell sequence of BA has already been reported, few reports concerning neutrophil has been revealed. Single-cell RNA-sequencing on neutrophil of BA was conducted on rotavirus-induced mice model (15). Here, we used scRNA-seq to analyze PBMC from children with BA, NK&T, neutrophil and endothelia were analyzed. These findings enhanced our understanding of BA pathogenesis and may facilitate the identification of novel immune targets for the effective management of BA.

Liver immune profiling in BA was disorganized and contribute to pathogenesis such as inflammation, fibrosis, biliary atresia, bile plug, etc. Genes in CD4T cells were enriched for function of inflammation, cytotoxicity and chemotaxi. Cytotoxic CD8^+^ T cell expansion was found and genes were characterized by high expression of IFN*γ* and granzyme (47). The oxidative stress response in NK cells is dysregulated in children with BA. ROS derives from cell metabolism, mitochondrial electron transport chain reaction and environment pollution such as drug and infection. Oxidative stress may cause DNA damage, protein oxidation, lipid oxidation, and is associated with cardiovascular disease, neurodegenerative diseases and metabolic disease. Hypoxia-inducible factor 1*α* (HIF1*α*) was highly expressed in human BA tissue, and HIF1*α* may result from hypoxia or oxidative stress, treatment in babies with BA with antioxidant N-acetyl cysteine (NAC) may reduce ROS production and inflammation (48), indicating BA is associated with oxidative stress. Our study illustrated that augmented expression of oxidative stress related genes in endothelia may be the result of inflammation of BA. Further, NK&T and neutrophils also present characteristics of oxidative stress, and these transcriptional features may be associated with the pathogenesis and severity of BA.

In our study, enrichment analysis was used to identify multiple pathways that promoted pathogenesis, and some highly enriched receptor ligand pairs were found. This suggests that there may be different specific key mechanisms of liver fibrosis in different diseases. BA is a heterogeneous, multifactorial disorder with unknown pathogenesis, which is characterized by liver inflammation and fibrosis. BA can cause damage to endothelia and interaction between endothelia and other sub-cell types contributes to the progress of BA. Previous report proved that macrovascular endothelial locates in the fibrotic niche of the liver in BA, and functions as ligand to correlates with cholangiocytes via TNFSF12_TNFRSF12A pairs and may contributes to the fibrosis process in BA (14). Other studies also proved that decreasing epithelial proliferation may facilitate alleviating BA phenotype (40). Using endothelia as the ligand cell type, we observed that several interactions were enhanced in BA, with CXCL12-CXCR4 showing increased interactions from endothelia and other immune cell subtypes. CXCR4 is a rhodopsin like G protein coupled receptor composed of 352 amino acids, and the chemokine CXCL12 is the only ligand for this receptor (49). It was previously found that CXCR4 was widely expressed in tumor (50, 51). Recently, accumulating evidence confirmed that CXCL12/CXCR4 involved in pathological process in fibrosis, inflammation, immunity, and angiogenesis (43, 52, 53). CXCL12/CXCR4 have also been shown to improve fibrosis in many organs including the heart, liver (49). However, CXCL12/CXCR4 pair has not been mentioned in the process of onset and progression in biliary atresia, here, we identified that CXCL12/CXCR4 pair was identified between endothelial and other immune cells, such as NK&T, B, MP and neutrophil, this connection suggests that similar immune modulation pathways may be active and endothelial may recruits immune cell in the pathogenesis of BA, highlighting the functional relevance of this pair. Although we clearly detected significant upregulation of the CXCL12-CXCR4 axis in our single-cell sequencing cohort, no significant enrichment of this ligand-receptor pair was observed in the public single-cell dataset of BA. This discrepancy may be attributed to heterogeneity in clinical samples or differences in disease progression. Notably, the CXCL12-CXCR4 axis was further validated using multiplex immunofluorescence in additional samples, confirming its presence and suggesting that this axis likely plays a crucial role in the progression of BA.

In our study, HC subtype comprises hepatocyte and cholangiocytes, cholangiocyte was injured and proliferated in BA, and cholangiocyte interaction with other cell type contributes to the progress of BA (26). In our study, VEGFB/VEGFR1 pair was enhanced between HC and endothelial subtype, further analysis illustrate that interactions were widely exist between HC and central venous, HC and periportal, HC and veins. Expression of VEGFs and angiogenesis have been associated with biliary fibrosis in various biliary tract diseases, moreover, polymorphisms in the VEGF gene have been shown to be closely linked to susceptibility to BA (54, 55). These findings further support the critical role of the VEGF-VEGFR1 regulator*y* axis in the pathogenesis of BA, and our results indicated the importance of this pair in pathogenesis of BA and may be a new target for the treatment of biliary atresia. Developing drugs or treatment methods targeting this signaling pathway may help improve the prognosis of patients with BA. Multiple L-R pairs, showed significant interaction potential, and immune inhibitors targeting these L-R pairs might represent a potential strategy targeting pathogenesis of BA.

Interpretation of this study may be constrained by several limitations. Despite analyzing a relatively large number of cells, the study included only 2 BA patients and 1 healthy control. However, the high-quality data of the single cell transcriptome from the 3 children allows us to identify and elucidate characteristic of cell types and supplied valuable insight for understanding the mechanism of BA. Another point that requires clarification is the selection of control samples in our study. We selected histologically normal paracancerous liver tissue from pediatric patients with hepatoblastoma as the control group. We acknowledge that truly normal liver tissue would be a more rigorous control, and the use of paracancerous liver tissue in this study reflects both practical constraints and methodological precedent. Truly normal pediatric liver tissue is exceedingly scarce owing to ethical and clinical accessibility limitations. In hepatoblastoma single-cell transcriptomic studies, paracancerous liver tissue has been widely adopted as a comparator (6). We further acknowledge that a field "effect"—whereby adjacent histologically normal tissue may exhibit molecular perturbations due to paracrine signaling from the tumor—has been documented in adult solid tumors (56). However, these effects are typically local and distance-dependent, and have been characterized primarily using bulk rather than single-cell transcriptomic approaches.

In summary, our study presented a comprehensive analysis of the liver immune cell and endothelia landscape of BA. Although previous studies have illustrated the immune characteristic in liver of BA, our observation adds another layer to the complex immune feature as well as that of epithelial. Despite the limitation of a relatively small sample size, the findings in this study are exploratory and our data provides a valuable resource and critical mechanistic insights for deepening our understanding of immune responses and cell interaction of BA, this understanding lays the groundwork for developing targeted interventions that aim to modulate inhibitory pathways and transcriptional regulation in patients.

## Data Availability

The original single-cell RNA sequencing data generated in this study have been deposited in the NCBI Gene Expression Omnibus (GEO) database under accession number GSE315212 and are publicly available at https://www.ncbi.nlm.nih.gov/geo/query/acc.cgi?acc=GSE315212. The publicly available single-cell RNA sequencing dataset GSE176189, which was used for additional analyses in this study, can be accessed at https://www.ncbi.nlm.nih.gov/geo/query/acc.cgi?acc=GSE176189.
